# Examining Associations Between State-Level Transgender Policies and Transgender Health

**DOI:** 10.1089/trgh.2018.0031

**Published:** 2018-12-26

**Authors:** Steve N. Du Bois, Wren Yoder, Arryn A. Guy, Kelly Manser, Stephen Ramos

**Affiliations:** Department of Psychology, Illinois Institute of Technology, Chicago, Illinois.

**Keywords:** transgender, gender nonconforming, health, policy, mental health, health care utilization

## Abstract

Compared with their cisgender counterparts, transgender individuals face both structural-level discrimination and health disparities across health domains. We used recent population-level data to examine associations between state-level policy ratings regarding transgender people and transgender health (poor physical and mental health days, health behaviors, and health care utilization). We hypothesized more inclusive and protective state-level policies would predict better health of transgender individuals. The sample (*N*=1116) was approximately half white (*n*=572, 51.2%) and half transgender women (*n*=551, 49.3%). More transgender-inclusive and protective state-level policies predicted better health in three of four health domains. Policy and research implications are discussed.

## Introduction

The term transgender describes individuals whose current gender identity or gender expression differs from their natal sex.^1^ In the United States, 1.3% of individuals, ages 13 and older, or 1.5 million people identify as transgender.^2^ Compared with their cisgender counterparts, transgender people are vulnerable to experiencing structural-level discrimination and poor mental and physical health. These co-occurring disparities can be understood through the Stigma-Sickness Slope (SSS) model of transgender vulnerability. This model asserts that stigmatized people—those believed to be less worthy than others—are marginalized by society. This societal marginalization is linked to poor health outcomes in multiple domains, for example, physical health, mental health, health behaviors, and health care access.^3^ Stigmatizing beliefs at the structural level can shape prejudiced policy as well as legitimize and exacerbate stigmatizing beliefs at the societal level, facilitating the marginalization and poor health of transgender individuals.^3^

Structural-level discrimination is a social determinant of health and is characterized by uneven distribution of power, rights, and resources.^4^ Research indicates that the majority of transgender individuals report experiencing structural-level discrimination, for example, past-year public accommodation discrimination.^5^ More broadly, public policy (e.g., institutional rules, state- and federal-level laws) can discriminate against transgender individuals in numerous ways. These include housing and employment antidiscrimination laws excluding transgender individuals from their list of protected populations,^6^ or proposed laws banning transgender individuals from the armed forces.^7^

Overall, compared with cisgender people, transgender people face the dual burden of more health concerns and more difficulty receiving adequate affordable care for those concerns. Transgender individuals report a higher prevalence of HIV, rates of depression, and number of suicide attempts.^3^ They also report more frequent engagement in risky health behaviors, for example, smoking and alcohol use.^8–10^ Concurrently, transgender individuals are less likely to access and utilize medical care, to complete medical tests, and more likely to be denied treatment when sought.^3^

Associations between structural-level discrimination and individual health have been explored more among lesbian, gay, and bisexual (LGB) than transgender individuals. Extant work indicates that LGB-related state-level policies are associated with LGB individuals' health. For example, Hatzenbuehler et al. conducted multiple studies utilizing data from the National Epidemiologic Survey on Alcohol and Related Conditions. They found that living in states without state-level policies protecting LGB individuals from hate crimes and sexual orientation-based employment discrimination predicted stronger associations between LGB status and psychiatric disorders than did living in states with such protections.^11^ In a different study, they found significant increases in psychiatric disorders among LGB individuals after states implemented policies banning marriage equality, but not among LGB individuals living in states that did not implement bans.^12^ Other work supports the idea that state-level, legal recognition of same-sex marriage may reduce health differentials between LGB and heterosexual individuals. For example, in a Californian sample, psychological distress was highest among LGB individuals not in legalized relationships but was not significantly different between heterosexuals and LGB individuals in same-sex marriages.^13^

In this study, we aim to extend previous work on policy and sexual and gender minority (SGM) health, by testing whether state-level policies regarding transgender individuals relate to the health of those individuals. We use data from two sources: the 2016 Behavioral Risk Factor Surveillance System (BRFSS)—the population-level data source of our large sample; and the Movement Advancement Project (MAP), an SGM advocacy group and think tank that provides our independent variable.^14^ Specifically, MAP assigns each state a “policy tally” based on four transgender-specific policy criteria: protection from discrimination in schools, ability to change name and gender on identifying documents, health care access, and protection from discrimination. We hypothesize that more inclusive and protective state-level transgender policies will relate to better health of transgender individuals in those states.

## Methods

### Participants and procedures

Data were extracted from the 2016 BRFSS, a yearly telephone survey of U.S. states, Guam, the U.S. Virgin Islands, Washington, D.C., and Puerto Rico. The BRFSS assesses the health of individuals, 18 or older, not living in an institutionalized setting, and who have access to a home/mobile telephone. The Centers for Disease Control and Prevention oversees data collection and provides a comprehensive BRFSS description at www.cdc.gov/brfss

Only 26 of the 54 participating BRFSS states/territories included the *Sexual Orientation and Gender Identity* (SOGI) module in their 2016 data collection. This module includes one item assessing sexual orientation and another assessing gender identity. Therefore, these data represent only states/territories and transgender individuals in states/territories that utilized this module.

### Measures

#### Sociodemographic variables

The BRFSS collects data on race, age, annual income, number of children, education, and other sociodemographics. Transgender identity was assessed via the item, “Do you consider yourself to be transgender? Yes, transgender, male-to-female; Yes, transgender, female-to-male; Yes, transgender, gender nonconforming; No, don't know/not sure; Refused.” Respondents who self-reported “Yes…” on this question were included in our sample.

#### State-level gender identity policy tally

Gender identity policy tally (GIPT) was operationalized using policy tally scores from the SGM advocacy group MAP. MAP calculates GIPT scores for each U.S. state, plus Washington, D.C. These scores incorporate 35 types of laws, including marriage/relationship rights, parenting rights, nondiscrimination laws, protection of SGM youth, overall health/safety, and ability to correct listed gender on personal documentation.^14^ Each pro-transgender law increases GIPT by one point; each discriminative law decreases GIPT by one point. Fractions of a point may be applied if a policy's impact is not statewide. Thus, the higher overall GIPT value indicates more legal protection of transgender rights, and a negative tally (<0) indicates a policy profile with adverse implications for transgender individuals. As of February 2017, GIPT scores ranged from −4.50 (Georgia) to 16.00 (California). Mean value was 3.53 (SD=6.01); median was 0.75.

#### Health

We assessed four domains of transgender health—physical health, mental health, health behaviors, and health care utilization—over the past 30 days. We selected these domains for three reasons: First, the SSS model of transgender vulnerability highlights each as a point of health vulnerability among transgender individuals.^3^ Second, empirical findings indicate that each relates to health policy.^11,12^ Third, in each domain, transgender individuals report disparities compared with cisgender counterparts.^3,8–10^

Regarding specific variable selection, for three of four health domains, we used BRFSS single-item, precalculated summary variables that represented data from multiple survey items. We selected the items “number of days physical health not good” (physical health), “number of days mental health not good” (mental health), and “length of time since last routine checkup (within past year; past 2 years; past 5 years; 5 or more years ago; health care utilization).” For the remaining health domain—health behaviors—we selected two items reflecting relatively prevalent and adverse behaviors within this population, alcohol use^8^ and smoking.^9^ One item assessed “average alcoholic drinks per day” and another assessed current smoking status (smoker, nonsmoker). Participants provided specific values for the physical health, mental health, and alcohol questions.

### Analytic plan

Analyses were conducted using SPSS version 24.0. Sample characteristics were calculated using descriptive statistics. Individual linear regressions were used to test the effect of states' GIPT scores on health variables. Logistic regression was run for the smoking variable, given its dichotomous nature. For each regression, we controlled for sociodemographic variables known to relate to our health variables—number of children in the home, education, income, race, and age.^15,16^ To account for increased type I error risk due to running multiple analyses, we used an alpha of 0.01 to determine significance.

Sample data were weighted using the BRFSS variable _LLCPWT, as instructed in the BRFSS module data for analysis. In addition, to adjust for the impact of weights on the standard errors in SPSS, researchers performed a normalization procedure: multiplying the weight (in this case _LLCPWT) by (Unweighted *N*)/(Weighted *N*).^17^

## Results

### Participants

The U.S. adults (*N*=486,303) provided data through the 2016 BRFSS. Sample data include transgender-identified individuals (*N*=1116) in the 26 states/territories that both included the SOGI BRFSS module, and for which MAP provided a policy tally. Table 1 presents sample demographics. Study participants were 49.3% (*n*=550) transgender women, 28.3% (*n*=316) transgender men, and 22.4% (*n*=250) gender nonconforming. Participants were ages 18–80 (*M*=42.3; SD=18.2).

**Table 1.** Participant Characteristics as a Percentage of the Sample

	*N*	%
Race		
White	572	51.2
Black	170	15.3
American Indian or Alaskan Native	15	1.3
Asian	60	5.3
Native Hawaiian or other Pacific Islander	5	0.5
Other	6	0.5
Multiracial	31	2.7
Hispanic	246	22
Don't know/not sure/missing	12	1.1
Sexual orientation		
Straight	658	58.9
Lesbian or gay	145	13
Bisexual	173	15.5
Other	74	6.6
Don't know/not sure	25	2.2
Missing	41	3.7
Education		
Did not graduate high school	284	25.5
Graduated high school	339	30.3
Attended college or technical school	359	32.2
Graduated from college or technical school	124	11.1
Don't know/not sure/missing	10	0.9
Income		
Less than $15,000	230	20.6
$15,000 to less than $25,000	184	16.5
$25,000 to less than $35,000	147	13.2
$35,000 to less than $50,000	99	8.9
$50,000 or more	265	23.8
Don't know/not sure/missing	191	17.1
Children in household		
No children in household	713	63.8
One child in household	199	17.8
Two or more children in household	187	16.8
Don't know/not sure/missing	18	1.6

### Hypothesis testing

Table 2 presents means and standard deviations for health variables, and regression results. Overall, a higher GIPT (more inclusive and protective state-level policy) significantly predicted fewer recent poor mental health days, fewer average alcoholic drinks per day, and a shorter length of time since last health care checkup. The number of recent poor physical health days and reported smoking behavior did not vary by GIPT, although notably the mean number of poor physical health days in the last 30 was high (11.97, SD=11.02).

**Table 2.** Descriptives, Missingness Correlations, and Health Variables Predicted by Gender Identity Policy Tally

Dependent variable	*M* (SD)	Pearson's *R*	Omnibus *F* test	df	*R*^2^	Δ*R*^2^	*B*	SE	β	*t*	95% CI
Physical health	11.97 (11.02)	−0.10^**^	8.69^**^	4.394	0.08	0.00	−0.03	0.08	−0.02	−0.42	−0.19 to 0.12
Mental health	14.65 (10.67)	−023^**^	10.48^**^	4.365	0.1	0.04^**^	0.32	0.08	0.20	4.06^**^	.0.16 to 0.47
Time since last checkup	1.62 (1.07)	−0.075^*^	18.92^**^	4.887	0.08	0.005^*^	−0.01	0.005	−0.07	−2.08^*^	−0.02 to −0.01
Alcohol	2.70 (9.55)	0.04	4.62^**^	4.874	0.02	0.01^**^	−0.15	0.05	−0.11	−3.17^**^	−0.24 to −0.06
Smoking	—	0.14^**^	0.32	1.8	—	—	−0.01	0.01	0.99	0.324	—

All regression analyses had number of children in the household, income, and age entered in step 1. Pearson's *R* is the point biserial correlation between missing data for each dependent variable and GIPT. Omnibus *F* test represents the significance test for the overall model (i.e., demographics at step 1 and GIPT at step 2). *R*^2^ is the proportion of variance accounted for by the model. Δ*R*^2^ is the increase in *R*^2^ after GIPT was added to the model in step 2. *B* is the regression coefficient for GIPT (e.g., a 1-point increase in GIPT predicted a 0.11 decrease in alcohol use). SE is the standard error for each regression coefficient. β is the standardized regression coefficient. *t* represents the significance test for GIPT as an individual predictor in the overall model.

Bold indicates the variable was missing 56–67%.

^**^ *p*<0.001; ^*^p<0.05.

CI, confidence interval; GIPT, gender identity policy tally.

During analyses, two variables emerged as having relatively low sample sizes due to a high proportion of missing data (56–67%). We ran *post hoc*, point biserial correlations to test for patterns of missing data (i.e., associations between missingness [missing vs. nonmissing] and GIPT), among all dependent variables. Table 2 reports these correlations. GIPT was significantly negatively correlated with missing data for length of time since last checkup, number of recent poor physical health days, and number of recent poor mental health days. That is, more protective/inclusive policies related to less missing data. GIPT was significantly positively correlated with missing data for smoking behavior, such that more protective/inclusive policies related to more missing data. GIPT did not relate to missing data for alcohol use.

## Discussion

Extant work indicates transgender individuals face both structural-level discrimination and multiple health disparities, and that these may be related.^3^ In this study, we used population-level data to examine whether structural-level inclusion, represented by state-level policies regarding transgender individuals, related to the health of transgender individuals in those states. More structural-level transgender inclusion, for example, protection from discrimination in schools and ability to change name and gender on identifying documents, predicted better mental health, less alcohol consumption, and more recent health care utilization among transgender individuals. State-level policy regarding transgender individuals did not predict smoking behavior or physical health.

Our findings can be understood through the SSS model, which asserts that transgender individuals may experience stigma associated with policies that explicitly exclude, or do not protect, them. This stigma could relate to poor health directly,^18^ and/or through a potential mediator such as societal marginalization, which itself relates to negative health behaviors such as problematic alcohol use,^19^ and increased likelihood of delaying needed health care or avoiding routine care.^5^ In either case, the associations between unfavorable state-level policies and poor mental health, greater alcohol consumption, and less recent health care utilization among transgender individuals characterize the “sickness” portion of the SSS model.^3^ While this interpretation reflects a protection perspective, that is, inclusive policies may protect transgender people from poor health, selection effects also may explain our findings.^20^ Perhaps healthier transgender individuals are “selecting into” (here, moving to) states with more favorable transgender policies.

Representing the sociopolitical relevance of this topic, numerous states recently have passed laws providing transgender people equal protection in employment, housing, and education.^21^ While empirical work has not assessed fully whether these policies relate to, or causally influence, the lives of transgender people, studies in related populations (e.g., LGB individuals) suggest that changes to structural-level factors such as state-level policies can improve the health of stigmatized groups.^11,13,22,23^ We encourage additional research on this topic, specifically temporal designs testing changes in transgender health as policies are implemented, removed, or changed.

Our findings have implications for both transgender research and transgender health. First, regarding transgender research, these findings demonstrate the importance of continuing to collect population-level data in this and other SGM populations. Such data are imperative to continue testing policy-health associations. Related, more states/territories should include the BRFSS SOGI survey module, and this module should be expanded to ask more than two relatively simple questions. Other population-level surveys should keep or move toward including items assessing gender identity. Our *post hoc* analyses indicated that the amount of missing data for physical health, mental health, and health care utilization was significantly correlated with state-level policy, meaning some transgender health data are relatively lacking in states with less inclusive/protective policies. This phenomenon is problematic insofar as it may facilitate the invisibility (at best) and erasure (at worst) of transgender health disparities. Regarding transgender health, our findings provide preliminary evidence of the health correlates of protective, inclusive transgender policies. Implementing more of these may relate to decreased stigma and improved health in this vulnerable population. Simultaneously, our findings corroborate past work stating that, independent of policy, high smoking prevalence and poor physical health persist among some SGM subgroups, and thus warrant additional attention.^9,13^

Study limitations include use of self-report data, although generally self-reported health correlates with objective health data.^24^ Risk for type I error was inflated by multiple comparisons; however, a 0.01 significance level partially addresses this. Analyses were cross-sectional, precluding causal interpretations. Many individuals and states were not represented in these analyses, and missing data were high for some health variables; however, use of a large, national sample permits relatively high generalizability.

Overall, the present study provides preliminary evidence that certain domains of transgender health may relate to state-level legal protections. Findings add to extant work that illuminates associations between structural equality and health among transgender individuals, encouraging continued research and sociopolitical focus on this vulnerable population.

## Abbreviations Used

BRFSS: Behavioral Risk Factor Surveillance System
CI: confidence interval
GIPT: gender identity policy tally
LGB: lesbian, gay, and bisexual
MAP: Movement Advancement Project
SGM: sexual and gender minority
SOGI: *Sexual Orientation and Gender Identity*
SSS: Stigma-Sickness Slope
